# Supplementary material for: SARS-CoV-2 seroassay optimization and performance in a population with high background reactivity in Mali
Source: medRxiv. 2021 Mar 12:2021.03.08.21252784. Preprint. [Version 1] doi: 10.1101/2021.03.08.21252784 (PMC7987042; doi:10.1101/2021.03.08.21252784)
Supplement: 1 [file NIHPP2021.03.08.21252784-supplement-1.pdf]

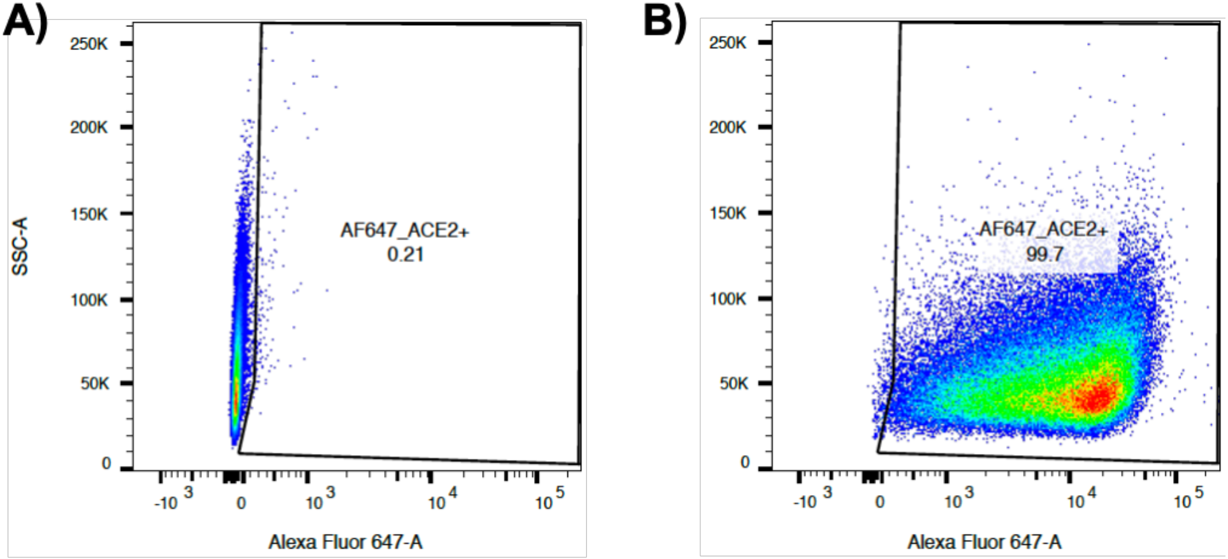

Supplementary Figure 1: Confirmation of BHK-21 cell ACE2 expression by flow cytometry using anti-hACE2 AlexaFluor 647-conjugated antibody. A) BHK-21 control B) BHK-21 BH-hACE2

medRxiv preprint doi: https://doi.org/10.1101/2021.03.08.21252784; this version posted March 12, 2021. The copyright holder for this preprint (which was not certified by peer review) is the author/funder, who has granted medRxiv a license to display the preprint in perpetuity.
This article is a US Government work. It is not subject to copyright under 17 USC 105 and is also made available for use under a CC0 license.

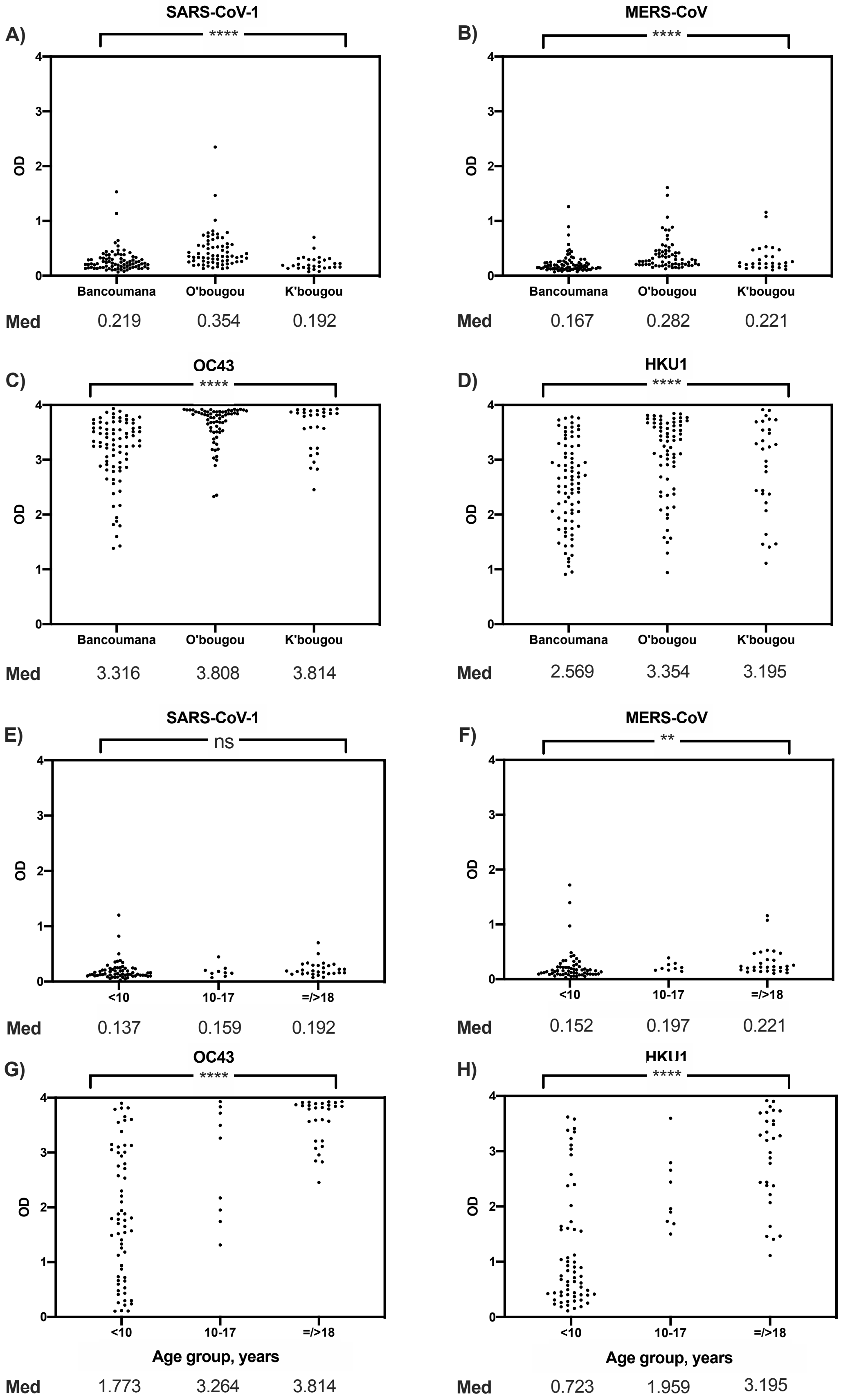

Supplementary Figure 2: Other betacoronavirus spike protein reactivity by site (age=/>18 years) in COVID-19 naïve Malian samples. A) SARS-CoV-1 B) MERS-CoV C) OC43 D) HKU1. SARS-CoV-2 antigen reactivity by age group (Kalifabougou site) E) SARS-CoV-1 F) MERS-CoV G) OC43 H) HKU1.

Med: median OD value, OD: optical density, RBD: receptor binding domain, NCP: nucleocapsid protein, O'bougou: Ouelessebougou site, K'bougou: Kalifabougou site

**** represents p<0.0001, ** p=0.001-0.01, ns represents p>0.05 using Kruskal-Wallis test.

Supplementary Table 1: Positive control cohort population characteristics

**Sex [male] (%, n)**	74% [17/23]
**Age, years (median [range])**	35 [7-74]
**Time since diagnosis, days (median [range])**	181 [27-270]
**Severity (%, n)**	
Asymptomatic	17% [4/23]
Mild (no oxygen)	57% [13/23]
Mod-severe (supplemental oxygen)	9% [2/23]
Critical (ventilator)	13% [3/23]
**Symptoms (%, n)**	
Asymptomatic	17% [4/23]
Paucisymptomatic	26% [6/23]
Symptomatic	57% [13/23]

Disease severity categorized using WHO stratification criteria (Clinical Management of COVID-19 WHO, May 27 [18])

medRxiv preprint doi: https://doi.org/10.1101/2021.03.08.21252784; this version posted March 12, 2021. The copyright holder for this preprint (which was not certified by peer review) is the author/funder, who has granted medRxiv a license to display the preprint in perpetuity. This article is a US Government work. It is not subject to copyright under 17 USC 105 and is also made available for use under a CC0 license.

Symptoms categorized as asymptomatic, paucisymptomatic (one symptom), or symptomatic (two or more symptoms.)

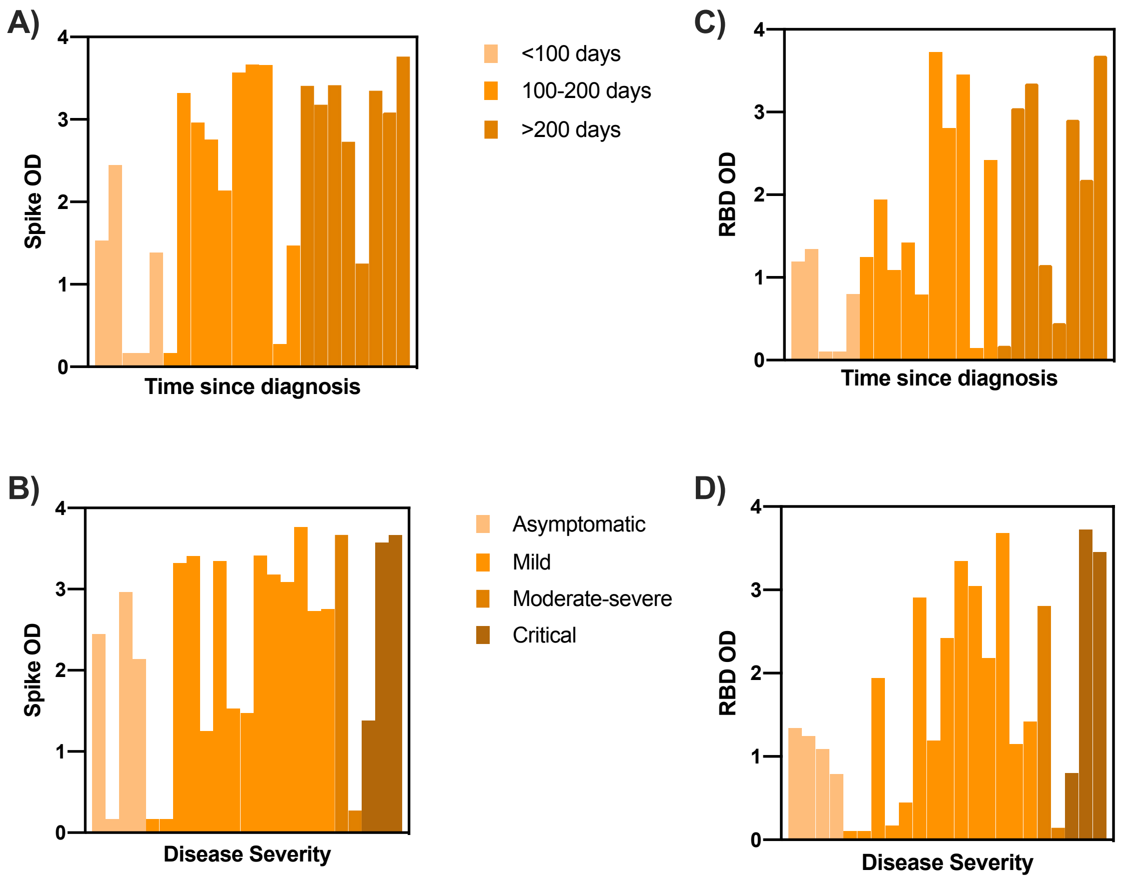

Supplementary Figure 3: Positive control cohort population reactivity to SARS-CoV-2 spike protein by ELISA according to: A) time since diagnosis and B) disease severity. Reactivity to RBD by ELISA according to: C) time since diagnosis and D) disease severity.

OD: optical density, RBD: receptor binding domain

Disease severity categorized using WHO stratification criteria (Clinical Management of COVID-19 WHO, May 27 [18])
